# Editorial: Smart nanomaterials for biosensing and therapy applications, volume II

**DOI:** 10.3389/fbioe.2024.1387969

**Published:** 2024-04-19

**Authors:** Jing Liao, Miaomiao Yuan, Ziqiang Xu, Youbin Zheng, Zhe Wang, Qitong Huang

**Affiliations:** ^1^ Key Laboratory of Prevention and Treatment of Cardiovascular and Cerebrovascular Diseases of Ministry of Education, Key Laboratory of Tissue Engineering of Jiangxi Province, Key Laboratory of Biomedical Sensors of Ganzhou, School of Medical and Information Engineering, School of Pharmacy, Science Research Center, Gannan Medical University, Ganzhou, China; ^2^ Precision Research Center for Refractory Diseases in Shanghai General Hospital, Shanghai Jiao Tong University School of Medicine, Shanghai, China; ^3^ School of Materials Science and Engineering, Hubei University, Wuhan, China; ^4^ Department of Electrical Engineering and Electronics, University of Liverpool, Liverpool, United Kingdom; ^5^ School of Biology and Agricultural Engineering, Jilin University, Changchun, China

**Keywords:** smart nanomaterials, biosensing, nanozyme, cancer therapies, intelligent drug-delivery system, early-diagnosis

Early diagnosis and effective treatment of diseases are key to improving people’s health levels (Yi et al., 2022; Zhang et al., 2022; Zheng et al., 2022; Yuan et al., 2023; Liu et al., 2024). However, there are still some problems in early diagnosis and efficient treatment, such as: 1) how to more sensitively measure disease markers to achieve early diagnosis of diseases, 2) how to make drugs effectively reach the lesion site and achieve efficient treatment? The effective resolution of the above problems will greatly promote the development of life sciences and medicine. Smart nanomaterials have been widely used in biomedical engineering and biotechnology due to their excellent physical, chemical, and biological properties, especially in the fields of biosensors and therapies (Huang et al., 2020; Chen et al., 2021a; Chen et al., 2021b; Huang et al., 2022; Yang et al., 2023; Luo et al., 2023; Thatte et al., 2023). Therefore, smart nanomaterials are expected to be used for early diagnosis of diseases and efficient treatment of diseases.

In order to present the most advanced research in this field, we have launched a Research Topic on “Smart Nanomaterials for Biosensing and Therapy Applications” in the “Frontiers in Bioengineering and Biotechnology”. This Research Topic has been published in two Research Topic. The first Research Topic has a total of 21 articles published in this Research Topic, including one editorial article, 11 original research articles, seven review articles, and two mini review articles. The second Research Topic has 10 articles published in this Research Topic, including eight original research articles, one review article, and one mini review article. The main content of the second Research Topic includes the application of smart nanomaterials in fields such as fluorescent probes, electrochemical sensors, tumor therapy, *Parkinson’s* disease treatment, and so on.

As is well known, high-performance early-diagnosis’ methods are key to disease prevention (Mei et al., 2022a; Mei et al., 2022b; He et al., 2022; Liao et al., 2023; Pourmadadi et al., 2023). Fluorescent probes and electrochemical sensors have played important roles in early disease detection. Chen et al. successfully prepared a near-infrared (NIR) fluorescent probe QSN based on a quinolone scaffold, which has strong photostability, large Stokes shift, and cell membrane targeting ability. After transplantation, human neural stem cells labeled with QSN are retained in the striatum of the mouse brain for at least 6 weeks, indicating the excellent application prospects of QSN in tracking ultra long term transplanted cells. As a member of smart nanomaterials, metal-organic frameworks/metal nanoparticles (MOFs/MNPs) have excellent chemical, physical, and biological properties, and have been widely used in electrochemical sensors. Jiang et al. reviewed the application of MOFs/MNPs-based composite materials in electrochemical sensors, providing some reference for the future development of electrochemical sensors based on MNPs/MOFs smart nanomaterials.

Currently, cancer is one of the deadliest diseases, accounting for one-sixth of global deaths, and it is an urgent health Research Topic that requires attention (Arnold et al., 2022; Song et al., 2023; Yang et al., 2023). Traditional methods for treating cancer include surgical resection, radiation therapy, and chemotherapy (CDT). However, traditional treatment methods have not been able to cure cancer well, so there is an urgent need to improve traditional methods or combine them with other methods to achieve cancer treatment. The emergence of smart nanomaterials not only promotes the development of traditional cancer methods, but also brings some new treatment technologies, such as photothermal therapy, photoacoustic therapy, photodynamic therapy (PDT), and so on, which will promote the development of cancer treatment. The second Research Topic of this Research Topic also published some latest achievements on the application of smart nanomaterials in cancer treatment.

Cisplatin (CDDP) drugs can act by interacting with DNA, leading to DNA damage and subsequent cell apoptosis, thereby achieving cancer treatment. However, the presence of intracellular PARP1 (Poly (ADP-ribose) polymerase 1) reduces the anti-cancer efficacy of CDDP by repairing DNA strands. Olaparib (OLA) enhances the accumulation of DNA damage by inhibiting its repair, effectively enhancing the sensitivity of CDDP chemotherapy and improving treatment outcomes. However, the CDDP and OLA drugs suffer from poor water solubility and limited tumor targeting ability. To overcome the above difficulties, Zhang et al. proposed the self-assembly of CDDP and OLA through hydrogen bonding to form stable and uniform nanoparticles. The modification scheme can improve the water solubility of two drugs, eliminate the need for exogenous carriers, and achieve targeted delivery to the tumor site through enhanced permeability and retention effect. These enhanced functions improve characteristics, promote cancer cell apoptosis, and minimize harmful effects on normal cells. The acidic tumor microenvironment disrupts hydrogen bonds, leading to the release of free CDDP and OLA. CDDP has the ability to induce DNA damage and initiate the process of cell apoptosis, while OLA enhances the anti-cancer sensitivity of CDDP by inhibiting PARP1 activity, thereby reducing the repair of damaged DNA and improving the efficacy of tumor treatment.

The construction of nanomedicine systems has always been a key factor affecting the effectiveness of nanomedicine in tumor treatment (Chen et al., 2022; Zhou et al., 2022; Wang et al., 2023a; Wang et al., 2023b). Zhang et al. successfully summarized the advantages and disadvantages of nanomedicine systems currently used for the treatment and diagnosis of esophageal cancer, and proposed multiple excellent suggestions for the development of nanomedicine systems in the future. Lv et al. have successfully prepared Fe@PCN-224@hyaluronic acid smart nanomaterial and used the smart nanomaterial to load sulfasalazine drug for the combined treatment of tumors with PDT and CDT (Lv et al.). This smart nanomaterial can effectively regulate mitochondria, reduce the tolerance of tumor cells to combination therapy, induce tumor cell apoptosis, and accelerate cancer treatment. Wang et al. designed a novel reactive oxygen species-sensitive polymeric prodrug micelle (Ce6@PTP/DP) with high drug-loading capacity and self-amplified drug release. The multidrug delivery system comprised three parts: a ROS-sensitive polymeric prodrug methoxyl poly (ethylene glycol)-thioketal-paclitaxel (mPEG-TK-PTX, PTP), DSPE-mPEG (DP), and a traditional photosensitizer of chlorin e6 (Ce6). The Ce6@PTP/DP prodrug micelles exhibit good colloidal stability and biocompatibility, with PTX and Ce6 loading amounts reaching 21.7% and 7.38%, respectively. Under light irradiation, ingested by tumor cells Ce6@PTP/DPmicelles can generate sufficient ROS, which can not only achieve photodynamic therapy and inhibit tumor cell proliferation, but also trigger local PTX release and methoxy polyethylene glycol by breaking down the thiol ketone bridge bond between PTX. In addition, compared to single drug loaded micelles, light triggered Ce6@PTP/DP micelles exhibit self amplifying drug release and significantly greater inhibition of HeLa cells growth. Therefore, the Ce6@PTP/DP micelles represent an alternative for realizing synergistic chemo-photodynamic therapy. Wang et al. utilized baicalin (BA) and polyethyleneimine (PEI) through condensation reaction to prepare BA-PEI nanocomposites with effective gene delivery and perfect transfection performance, including charge regulation, hydrophobic modification, and functional modification. BA-PEI nanocomposites can effectively load miR-34a, thereby constructing a new type of nanotherapy system. miR-34a can synergistically exert anti-tumor effects with BA to enhance the therapeutic effect, which provided a new method for gene therapy based on miR-34a.

In addition to being applied in cancer treatment, the application of smart nanomaterials in the treatment of other diseases has also received much attention. Li et al. constructed an antibacterial nanomaterial that combines photothermal effect and peroxidase like activity ZIF-8@PDA@PtNPs combines photothermal antibacterial and free radical antibacterial strategies to achieve efficient synergistic antibacterial activity (Li et al.). By *in-situ* polymerization, a polydopamine (PDA) layer with excellent photothermal effect was encapsulated on the surface of ZIF-8, and then platinum nanoparticles (PtNPs) with peroxidase like activity were grown on the PDA layer. The synergistic effect of the two produces efficient antibacterial activity: The obtained nano antibacterial agent not only efficiently catalyzes the generation of ROS from H_2_O_2_, causing damage to bacteria, but also converts the photon energy of near-infrared light into thermal energy to kill bacteria. Deng et al. have prepared a biological nanomaterial composed of Fibrinogen-Thrombin-Genipin, which can effectively repair defects in the annulus fibrosus (AF) of the scaffold in rats’ model, providing a therapeutic alternative for repairing AF tears (Deng et al.). Xu et al. constructed a ternary nanoenzyme PtCuSe smart nanomaterial as a cascade catalyst for the treatment of Parkinson’s disease (PD) (Xu et al.). This smart nanomaterial was used as a ROS scavenger both *in vitro* and *in vivo*, effectively alleviating oxidative damage and inflammatory reactions in nerve cells, and significantly alleviating behavioral and pathological symptoms in PD mouse models.

The application of intelligent nanomaterials in biosensing and therapy is still of great concern. Therefore, in order to get the latest achievements in this field, the Research Topic will launch the third Research Topic, entitled “Smart Nanomaterials for Biosensing and Therapy Applications, Volume III”, welcome to submit and share your latest research.
